# What factors predict ambulance prealerts to the emergency department? Retrospective observational study from three UK ambulance services

**DOI:** 10.1136/bmjopen-2024-097122

**Published:** 2025-03-07

**Authors:** Richard Pilbery, Fiona C Sampson, Esther Herbert, Steve Goodacre, Fiona Bell, Rob Spaight, Andy Rosser, Peter Webster, Mark Millins, Andrew Pountney, Joanne Coster, Jaqui Long, Rachel O’Hara, Alexis Foster, Jamie Miles, Janette Turner, Aimee Boyd

**Affiliations:** 1 Research and Development, Yorkshire Ambulance Service NHS Trust, Wakefield, UK; 2 SCHARR, School of Medicine and Population Health, The University of Sheffield, Sheffield, UK; 3 Yorkshire Ambulance Service NHS Trust, Wakefield, UK; 4 East Midlands Ambulance Service NHS Trust, Nottingham, UK; 5 West Midlands Ambulance Service NHS Foundation Trust, Brierley Hill, UK; 6 Leeds General Infirmary, Leeds, UK; 7 Clinical Directorate, Yorkshire Ambulance Service NHS Trust, Wakefield, UK; 8 South East Coast Ambulance Service NHS Foundation Trust, Banstead, UK

**Keywords:** Emergency Departments, STATISTICS & RESEARCH METHODS, ACCIDENT & EMERGENCY MEDICINE

## Abstract

**Objectives:**

Ambulance clinicians use prealert calls to advise emergency departments (ED) of the arrival of patients requiring immediate review or intervention. Consistency of prealert practice is important in ensuring appropriate ED response to prealert calls. We used routine data to describe prealert practice and explore factors affecting variation in practice.

**Design and setting:**

We undertook a retrospective observational study in three UK ambulance services using a linked dataset incorporating 12 months’ ambulance patient records, ambulance clinician data and emergency call data.

**Outcome measures:**

We used least absolute shrinkage and selection operator regression to identify candidate variables for multivariate logistic regression models to predict variation in prealert use, analysing clinician factors (role, experience, qualification, time of prealert during shift), patient factors (National Early Warning Score version 2, clinical working impression, age, sex) and hospital factors (receiving ED, ED handover delay status).

**Results:**

From the dataset of 1 363 274 patients conveyed to ED, 142 795 (10.5%) were prealerted, of whom 42 362 (30%) were for conditions with clear prealert pathways (eg, sepsis, stroke, ST-elevation myocardial infarction, major trauma). Prealert rates varied across and within different ambulance services. Casemix (illness acuity score, clinical diagnostic impression) was the strongest predictor of prealert use, but male patient sex, clinician role, receiving hospital and hospital turnaround delay at receiving hospitals were also statistically significant predictors, after adjusting for casemix. There was no evidence that prealert rates are higher during the final hour of shift.

**Conclusions:**

Prealert decisions are influenced by factors other than illness acuity and clinical diagnostic impression alone. Variation in prealert practice suggests that procedures and processes for prealerting may lack clarity and improved prealert protocols may be required. Research is required to understand whether our findings are reproducible elsewhere and why non-clinical factors (eg, patient gender) may influence prealert practice.

**STRENGTHS AND LIMITATIONS OF THIS STUDY**

- This study used routine data to describe prealert practice in three UK ambulance services to understand why patients receive prealerts and to explore potential factors affecting practice.
- The study incorporated a linked dataset of 1.3 million ambulance conveyances, linking electronic patient record data with clinician and hospital data to identify candidate variables for multivariate logistic regression models to predict variation in prealert use.
- The time period covered by the study included the second period of COVID-19 lockdown in the UK (January–March 2021), which potentially reduces the transferability of findings.
- The study was designed to be exploratory to identify what variables might predict the use of prealerts and may be affected by different coding criteria between the different ambulance services.

## Introduction

 Ambulance clinicians use prealert (prenotification) calls to inform receiving emergency departments (EDs) of the arrival of a critically unwell or rapidly deteriorating patient who they believe requires urgent review and/or time-critical treatment immediately on arrival. Prealerts enable the receiving ED to prepare for the patient’s arrival, including actions such as activating a trauma team, requesting specialist support, preparing specialist equipment or ensuring availability of a resuscitation bay.^1^ The use of prealerts in pathways for certain conditions (eg, stroke, ST-elevation myocardial infarction (STEMI), major trauma) is now well established, but prealerts are also recommended for other physiological criteria or specific conditions, or patients judged to be ‘seriously injured or critically ill’.^2^,^4^ In the UK, joint guidance developed by the Association of Ambulance Chief Executives (AACE) and Royal College of Emergency Medicine (RCEM) includes a number of physiological criteria and specific conditions that should be considered for prealert, for example, tachycardia ≥131 and STEMI.^2^ Prealerts can lead to earlier initiation of time-critical treatment, improved processes and better clinical outcomes for patients in specific patient groups including stroke, STEMI and sepsis.^5^,^12^

There are concerns that a lack of effective policies for prealerting may lead to inconsistent response and suboptimal patient care.^3^ Overuse or inappropriate use of prealerts may lead to patient safety risks due to EDs diverting resources from other critically ill patients.^1 6 7 13^ Concerns about prealerts being used to bypass ambulance queues outside overcrowded EDs have been raised, with suggestions that prealert thresholds may be related to how busy ambulance clinicians believe the ED may be, which is particularly problematic in the context of overcrowded EDs.^14 15^ Similarly, there are concerns about ‘prealert fatigue’ where overuse of prealert calls leads to ED staff placing less value on the prealert.^16^ Consistent and appropriate use of prealerting is, therefore, key to optimising care for patients, particularly in the context of increasing demand and overcrowded EDs.^3 17^

Risk adversity in ambulance clinician decision-making has been shown to be influenced by levels of experience, confidence and fear of blame.^18^ Given the lack of consistency in guidance at an organisation level, increasing concerns about litigation and pressures to meet ambulance handover targets, we hypothesise that prealert practice is affected by factors other than patient presentation and physiology.^19^ There is some evidence of disparities in prealert use for stroke or STEMI, with variation based on hospital, region and patient characteristics.^20^,^22^ This variation is likely to be higher for conditions that constitute high numbers of prealerts, yet have less clear diagnostic criteria or prealert pathways (eg, suspected sepsis, unspecified medical concern). Despite prealerts playing a key role in the transfer of care between ambulance and ED clinicians, and recognition of the burden that prealerts can place on already stretched EDs, understanding of which patients are prealerted and what factors may contribute to variation in practice is still limited.^22^

We used routine data to describe prealert practice in three of the 13 UK National Health Service (NHS) ambulance services to understand which patients are receiving prealerts and to explore potential factors affecting variation in practice. Specifically, we aimed to understand whether there were clinician, patient and/or hospital factors that influenced prealert practice beyond the patient’s presenting complaint. The three ambulance services covering a population of 15.5 million were selected due to their ability to provide electronic patient record (ePR) data and because they incorporated areas of high and low deprivation, urban/rural mix and diverse ethnic populations that are broadly representative of the population of England.

## Methods

### Study design and setting

In the UK, ambulance services are part of the UK NHS but organisationally independent from hospitals. Ambulance clinicians are paramedics and other clinical staff, such as ambulance technicians, who usually work autonomously without physician support but within clearly defined scopes of practice. If the ambulance clinician determines the need for a prealert, they typically phone a dedicated number in the ED and provide information in a structured way. The ED clinician then determines the hospital response and informs the ambulance clinician where they should bring the patient (eg, triage, resuscitation bay).

We developed a logic model for factors affecting prealerts based on an informal rapid review of the literature and stakeholder consultation with three UK Ambulance Service Research leads and the UK RCEM/AACE. The model assumes that prealert practice may be affected by clinician factors (role, experience, sex, time of prealert during shift), patient factors (age, sex, National Early Warning Score version 2 (NEWS2) used to measure acuity, clinical working impression), hospital factors (catchment ED, handover delay status at time of prealert) and journey time.

We obtained routine, retrospective data from three adjoining ambulance services in England, covering a total population of 15.4 million people with a wide urban/rural and demographic mix. The ambulance service sites were selected pragmatically, based on their high rates of ePR completion and accessibility with ePR usage rate between 90% and 100%.

### Analysis

We analysed 12 months’ ePR data for all 999 calls that resulted in an ambulance transporting a patient to a hospital ED between 1 July 2020 and 30 June 2021. We linked data relating to the receiving hospital, including daily statistics on ambulance handover delay status at the time of prealert from routine situation report (SITREP) data,^23^ and ambulance clinician and patient data for each incident (table 1).

**Table 1:** Potential predictors for multivariable logistic regression model

Hospital factors	
Journey time	Elapsed time in minutes from the ambulance leaving the scene to arriving at hospital.
Handover delay status	Proportion of ambulance arrivals where patient handover took >30 min.^23^
Hospital	Anonymised name of the hospital. Note: Prealerts to hospitals with no ED were excluded.
Ambulance clinician factors	
Clinical experience	Time in years that clinician has worked in a clinical role with the ambulance service. Only available for sites 1 and 2.
Role	Clinical role category: Senior clinician, paramedic, newly qualified paramedic (<2 years since qualification), non-registered clinician (eg, emergency medical technician, associate ambulance practitioner) and non-registered clinical support staff.Simplified role only available for site 3—paramedic/non-registered clinician
Sex	Male/female/other
Ethnicity	Office for National Statistics categories. Excluded due to poor completion.
Proportion of shift	Binary variable to indicate whether the calls were in the final hour of ambulance clinician’s shift. Unable to obtain for site 3.
Patient factors	
Age	Continuous variable
Sex	Male/female/transgender
NEWS2*	First and last NEWS2 score obtained from clinician recorded observations in the electronic patient record.
RCEM prealert criteria.	Binary variable denoting whether the patient met either the physiological and/or non-physiological criteria for a prealert (see online supplemental additional file 1)

* NEWS2 is an early warning score based on routine physiological measures that is used in UK ambulance services and hospitals as a measure of illness acuity.^31^

EDemergency departmentNEWS2National Early Warning Score version 2RCEMRoyal College of Emergency Medicine

We undertook univariate analysis to describe prealert practice, including patient characteristics and clinical information for all conveyances with and without a prealert to understand which patients and clinical conditions were prealerted. Variable selection for the multivariable logistic regression model was performed using least absolute shrinkage and selection operator (LASSO).^24^ The LASSO process begins with a full model of all potentially relevant predictors and simultaneously performs predictor selection and penalisation during model development to avoid overfitting. Potential hospital, clinician and patient predictors considered for the logistic regression model are listed in table 1.

We excluded cases from the regression model where the patient was transferred to the ED from another healthcare setting (interfacility transfers), or who were taken to the ED with a clinical working impression of STEMI or hyperacute stroke, to mitigate for cases who had prealerted as part of an established ED bypass pathway. We excluded patients under 16 years of age due to different physiological/NEWS2 thresholds.

Due to high levels of correlation between NEWS2 scores and individual physiological criteria, we only included NEWS2 (ie, not separate observations) in the final model. However, to account for presentations that may be prealertable but not identified by NEWS2 (eg, acute stroke) we also included patient presentations specified within the UK RCEM/AACE prealert criteria.^2^ To accomplish this, we developed a criterion comprising ambulance clinician working impression, documented interventions and physiological variables (online supplemental additional file 1).

We developed two clinician role categories. A simplified clinician role variable was available for all cases (paramedic, non-registered clinician). For sites 1 and 2 where more granular data were available, clinician roles were allocated one of five categories (senior clinician, paramedic, newly qualified paramedic (NQP), non-registered clinician and non-registered clinical support staff).

Where a calculated NEWS2 score was not available in the ePR data, we calculated the NEWS2 score from the available physiological variables. Missing data were imputed with the value zero, classifying missing as normal, unless 3 or more physiological variables were missing.

### Patient and public involvement

Our study patient and public involvement (PPI) group included people with lived experience of prealerts, either through being prealerted themselves or as a carer or family member of someone who has been prealerted. A member of the PPI group was a coapplicant on the study, and they attended all of the project management meetings where the research process and analysis were discussed. In addition, regular PPI group meetings were held to discuss the research, and the PPI group attended a 3-hour workshop where they were asked to comment on the findings.

## Results

We included 1 363 274 conveyances in the descriptive analysis after removal of 18 668 interfacility transfers. Baseline characteristics and characteristics of prealerts are presented in table 2. The dataset included 142 795 prealerts, with prealert rates by ambulance service of 8%–15%. Baseline populations were similarly matched for sex, but site 3 patients were older (table 3—see online supplemental additional file 2). Overall, 75% of clinicians were paramedics (including newly qualified and senior paramedics) who prealerted a higher proportion of conveyances than non-paramedics (11.0% vs 9.8%).

**Table 2:** Summary table of categorical variables relating to ambulance transports to hospital stratified by prealert (% prealerted for each category)

Characteristic	Site 1 prealert/total (%)	Site 2 prealert/total (%)	Site 3 prealert/total (%)	Total prealert/ total (%)
Overall	60 549/413 140 (14.7)	51 142//623 325 (8.2)	31 104/326 809 (9.5)	142 795/1 363 274 (10.5)
Simplified clinician role				
Paramedic	46 665/305 549 (15)	39 342/485 978 (8.1)	21 302/211 206 (10)	107 309/1 002 733 (11)
Non-paramedic	13 884/107 591 (13)	11 800/137 347 (8.6)	9802/115 603 (8.5)	35 486/360 541 (9.8)
Patient sex				
Male	31 550/196 484 (16)	26 922/298 979 (9.0)	16 263/157 058 (10)	74 735/652 521 (11)
Female	27 871/210 636 (13)	23 835/319 200 (7.5)	14 764/168 699 (8.8)	66 470/698 535 (9.5)
Not specified	0/0	385/5146(7.5)	53/691(7.7)	438/5837(7.5)
Transgender	15/163(9.2)	0/0	22/355(6.2)	37/518(7.1)
Working Impression (conditions with >5 k prealerts only)				
Sepsis	6705/10 138(66)	11 402/20 679 (55)	3372/4004(84)	21 479/34 821 (62)
Unspecified medical condition	5668/62 620(9.1)	8673/165 959 (5.2)	2470/48 208(5.1)	16 811/276 787 (6.1)
Acute stroke	5881/8308(71)	6417/13 812(46)	2571/5663(45)	14 869/27 783 (54)
Other working impression	5791/71 923(8.1)	3848/90 264(4.3)	2134/54 066(3.9)	11 773/216 253 (5.4)
COVID-19	3525/13 942(25)	4481/25 550(18)	928/4285(22)	8934/43 777 (20)
Respiratory problem	5839/26 628(22)	676/6479(10)	2070/15 144(14)	8585/48 251 (18)
Arrhythmia	2504/6740(37)	1530/10 335(15)	1970/8069(24)	6004/25 144 (24)
Lower respiratory tract infection	926/5059(18)	2321/19 489(12)	2532/13 287(19)	5779/37 835 (15)
Cardiac problem	1919/21 419(9.0)	701/22 097(3.2)	2687/34 571(7.8)	5307/78 087 (6.8)
Any RCEM prealert criteria triggered	40 437/98 149 (41)	40 250/145 989 (28)	22 536/80 461 (28)	103 223/324 599 (32)
Any RCEM physiological criteria triggered	28 990/79 956 (36)	29 027/117 873 (25)	18 911/71 907 (26)	76 928/269 736 (29)
Any RCEM non-physiological criteria triggered	22 566/34 267 (66)	24 722/57 871 (43)	8658/17 209 (50)	55 946/109 347 (51)

Further details are provided in online supplemental additional file 4.

RCEMRoyal College of Emergency Medicine

**Table 3:** Summary of multivariable logistic regression following LASSO variable selection for ambulance service prealert for sites 1 and 2

Term	OR (95% CI)
Newly qualified paramedic	1.3 (1.26 to 1.34)
Paramedic	1.11 (1.08 to 1.13)
Senior clinician	1.09 (1.03 to 1.15)
Male clinician	0.98 (0.96 to 1)
Proportion of hospital turnarounds exceeding 30 min	1.74 (1.6 to 1.9)
Patient age	1.01 (1.01 to 1.01)
Male patient	1.24 (1.22 to 1.26)
Patient presentation meets RCEM non-physiological prealert criteria	15.85 (15.52 to 16.18)
Site one ambulance service	1.94 (1.86 to 2.02)
Emergency departments (not designated as major trauma centres) 1–25 (see online supplemental additional file 4)	ORs between 0.38 (0.18 to 0.7) and 2.2 (2.11 to 2.3)
Major trauma centres 1–8 (see online supplemental additional file 4)	ORs between 0.81 (0.77 to 0.85) and 1.66 (1.58 to 1.74)

Further details are supplied in online supplemental additional file 4.

LASSOleast absolute shrinkage and selection operatorRCEMRoyal College of Emergency Medicine

We tabulated the ambulance clinician working impressions with the highest number of prealerts. The most common prealertable conditions were sepsis (34 821 prealerts), unspecified medical condition/other (28 584 prealerts), acute stroke (14 869 prealerts) and COVID-19/respiratory problem/lower respiratory tract infection (23 298 prealerts). Prealert rates varied between sites for different conditions.

Sepsis and unspecified medical conditions accounted for just over a quarter of prealerts (26.9%). Stroke and STEMI (which have clear prealert and ED bypass pathways in the UK) accounted for a further 13%. Major trauma constituted under 2% of prealerts, although this rose to 4% when incorporating trauma/head injury.

Summary table of ambulance transports to hospital stratified by prealert.

### Logistic regression

After excluding cases where the ED was bypassed, patients who were under 16 years of age or whose age was not reported, and clinical working impressions of stroke or STEMI, the final dataset included 1 129 087 records for analysis. Due to differences in availability of clinician-related data between the three sites, we were unable to include clinician-related data within a single dataset for all services. As a result, we created a multivariable logistic regression model utilising combined data from sites 1 and 2 (table 3). Table 3 presents the data including ‘ambulance service’ as a variable, which an analysis of variance test suggested was a more accurate model than when ambulance service was excluded (online supplemental additional file 3).

### Hospital factors

Journey time was not selected by the LASSO as a significant feature contributing to prealerts and so does not appear in the logistic regression. However, there was considerable variation in odds ratios (OR) between hospital sites. Major trauma centre-designated hospitals were generally associated with an increased odds of a prealert, although this was not universal (eg, MTC5, MTC6, MTC8). The variation was wider in the other EDs, with ORs ranging from 0.38 (95% CI 0.18 to 0.7, ED5) to 2.2 (95% CI 2.11 to 2.3, ED25). In addition, increasing hospital turnaround was associated with increased odds of making a prealert (1.74, 95% CI 1.6 to 1.9).

### Ambulance clinician factors

While the length of clinician experience was not associated with prealerts, NQP (typically those who have been registered for less than 2 years) had a higher OR than other clinicians (OR 1.3 95% CI 1.26 to 1.34 vs 1.11, 95% CI 1.08 to 1.13 for paramedics). Clinician sex did not appear to have a significant impact on prealerts and calls within the final hour of the shift were not selected by LASSO. Staff at site 1 were more likely to prealert than those at site 2 (OR 1.94, 95% CI 1.86 to 2.02).

### Patient factors

Although prealerted patients are older, this appears to be explained by case mix, and age appears to have a minimal impact on prealert decision (OR 1.01, 95% CI 1.01 to 1.01). Male patients had a higher OR than female (OR 1.24, 95% CI 1.22 to 1.26). Interestingly, NEWS2 was not selected as a feature for inclusion in the model by LASSO, but meeting the RCEM non-physiological criteria for a prealert was the most statistically significant predictor of making a prealert (OR 15.95, 95% CI 15.52 to 16.18).

## Discussion

Our analysis of over 1.3 million ambulance conveyances identified differences in prealert practice that were not attributable to case mix. We identified that prealert practice was affected by a combination of hospital, clinician and patient factors. Although many prealerts were for key prealertable conditions (sepsis, stroke, STEMI or trauma), around two-thirds of prealerts were for conditions that may require a higher level of clinical judgement when deciding whether to prealert. Within this analysis, receiving hospital and hospital turnaround status were key factors in prealert practice, suggesting that ambulance clinician concerns about anticipated response from the receiving ED may have an influence over decision making. There was some evidence that NQPs may prealert more than more experienced clinicians, although the size of the effect detected was small. We found no evidence that clinicians in the final hour of their shift were more likely to prealert.

Despite the impact and importance of prealerts on patient care, we have not identified other literature exploring prealert practice and factors affecting prealert rates for general populations, although several studies have reported on prealert practice for specific conditions where the benefit of prealert is more clearly defined. Within our PPI consultation workshop, our PPI group stated concerns about the levels of variation identified within guidance and within practice (as reported here), with concerns about the implications of variation on patients and their families. They felt that local variation needed to be reduced to a minimum, with more emphasis placed on national guidance.

Differences in practice between ambulance services suggest local protocols and priorities have an impact on prealert decisions. Boyd *et al* identified important differences in ambulance service guidance in the UK, with differences in physiological thresholds for prealert even for conditions with established care pathways, with services listing between 4 and 45 conditions as suitable for prealert.^19^ There was also variation in national ambulance and ED guidance regarding the criteria to determine whether a prealert is appropriate.^2^ In the USA, emergency medical services (EMS) criteria for prealerts are likely to vary, and practice appears to be dictated by the requirements of local EDs.^3 25^ O’Hara *et al* also identified that differences in prealert criteria and perceived receptiveness to prealerts differed between EDs and affected how ambulance clinicians made prealert decisions.^26^ Lin *et al* identified statistically significant differences in EMS prehospital notifications for stroke between hospitals and regions and concluded that disparities in EMS prenotification use occurred by state and geographic region.

Inconsistent prealerting within individual hospitals has been reported elsewhere, with Sheppard *et al* and Brown *et al* both identifying underalerting of patients with suspected stroke, reporting that prealerts were not consistently used in suspected stroke patients, with 27% of patients who were FAST positive not prealerted, and 22% of patients who met the local criteria for prealert not being prealerted.^6 7^

Other studies have identified differences in patient factors affecting prenotification for specific conditions. Blusztein *et al* identified male sex as an independent predictor of prenotification for STEMI.^27^ Lin *et al* found that female patients were less likely to receive EMS prenotification for stroke but also identified higher likelihood of EMS prenotification for younger patients, and significant ethnic disparities in prenotification, with an adjusted OR of prealert for black patients of 0.94 (CI0.92–0.97) compared with white patients.^28^ Sheppard *et al* did not identify any statistically different racial or sex differences in stroke prealerting, which could be due to the small overall sample (n=271).^7^

It is unclear whether differences identified within our study are due to case mix that was not detected within the model, implicit bias in practice or different presentation of symptoms. We were unable to explore racial differences due to poor reporting of ethnicity within the ePR. However, our study supports findings of previous studies that report disparities in treatment based on non-clinical patient characteristics and suggests that inequalities in care exist.^29^

Our findings also demonstrate that prealert decision-making is affected by clinician and contextual factors, with prealert decisions being affected by anticipated ambulance handover delay as well as clinical experience. Weyman and O’Hara similarly identified that clinician perceptions of personal vulnerability and organisational blame in the event of a wrong decision (eg, waiting in a queue) are likely to influence more risk-averse decision-making.^30^

### Limitations

The differences in prealert rates between the three ambulance services are likely due partly to organisational differences but also due to differences in rates of recording the prealert within the ePR. At site 1, prealert recording was mandated during the final 6-month period and, therefore, likely to provide an accurate estimate of true prealert recording rates. However, recorded prealerts did not increase during this period.

We are unclear whether the missing data are missing at random. Missing data may be more likely for sicker patients where the ePR is likely to be completed after patient handover. However, although prealert recordings may not be missing at random due to patient condition, this is unlikely to affect other results such as clinician role, receiving hospital or patient sex.

The difference in prealert rates between sites may be due in part to under-recording of prealerts, or due to differences in local protocol. Data suggest that sepsis prealerts are higher at site 3 than for other sites, which reflects the local protocol requiring prealert for any red-flag sepsis. However, it is not known whether this reflects genuinely higher prealerting rates or higher recording of prealerts.

This study was undertaken within three UK ambulance services, and although we feel that the areas covered by these three services are broadly representative of the population of the UK, transferability may be limited for settings outside the UK. However, given known recognised variation in practice and a lack of clear protocols within other settings, the level of variation identified within this study is likely to be found elsewhere. The time period for which data were collected included the second period of COVID-19 lockdown in the UK (January–March 2021), which reduces the potential transferability of findings. The proportion of prealerts due to COVID-19 or respiratory disease was likely higher within this dataset than in other years. However, this is unlikely to affect prealert practice for other conditions significantly.

We adjusted for casemix using the UK AACE/RCEM non-physiological criteria for a prealert. However, coding of this field required assumptions to be made, including the use of physiological parameters for some non-physiological criteria since working impression codes were either not available for certain prealertable presentations, or the severity of the patient presentation could not be determined by the working impression alone (eg, drug overdose, trauma, haemorrhage). This coding had to be undertaken on a service-by-service basis since provided fields and working impression codes differed between services. It is possible that this may lead to differences in categorisation in the model.

Differences in proportions of patients with sex labelled as ‘transgender’ or ‘not reported’ differed by ambulance service, suggesting that some ambulance services did not use the category ‘transgender’ within their coding and this field may not be reliable.

The analysis was exploratory in nature and not confirmatory. The aim was to explore what variables might predict the use of prealerts, with the aim to guide future research. Although the size of effect was different within the combined and separate models (which may be expected as these were different datasets), the different models did all show that clinical variables are the key predictors, with hospital factors, anticipated handover delay, patient sex and clinician role all being predictors.

## Conclusions

Considerable levels of variation in prealert practice across and within different ambulance services suggest that procedures and processes for prealerting may lack clarity, and improved prealert protocols may be required to reduce variation. While broad definitions such as ‘critically ill’ enable clinicians to use their clinical acumen to decide whether a patient requires a prealert, this may also enable overuse of prealerts and increase the risk of prealert fatigue. There is a need to understand the reasons behind the variation in prealert practice identified within this analysis.

## supplementary material

10.1136/bmjopen-2024-097122 online supplemental file 1

10.1136/bmjopen-2024-097122 online supplemental file 2

10.1136/bmjopen-2024-097122 online supplemental file 3

10.1136/bmjopen-2024-097122 online supplemental file 4

## Data Availability

Data are available on reasonable request.
