# Supplementary material for: What factors predict ambulance prealerts to the emergency department? Retrospective observational study from three UK ambulance services
Source: BMJ Open. 2025 Mar 7;15(3):e097122. doi: 10.1136/bmjopen-2024-097122 (PMC11891521; doi:10.1136/bmjopen-2024-097122)
Supplement: online supplemental file 2 [file bmjopen-15-3-s002.docx]

Summary table of continuous variables relating to ambulance transports to hospital stratified by pre-alert.

|  | **Site 1** | | | **Site 2** | | | **Site 3** | | |
| --- | --- | --- | --- | --- | --- | --- | --- | --- | --- |
| **Characteristic** | no pre-alert, N = 352,591^1^ | Pre-alert, N = 60,549^1^ | Overall, N = 413,140^1^ | no pre-alert, N = 572,183^1^ | Pre-alert, N = 51,142^1^ | Overall, N = 623,325^1^ | no pre-alert, N = 295,705^1^ | Pre-alert, N = 31,104^1^ | Overall, N = 326,809^1^ |
| **Ambulance journey time (mins)** | 17 (11, 24) | 14 (9, 21) | 17 (11, 24) | 16 (11, 23) | 12 (9, 18) | 16 (11, 23) | 18 (12, 27) | 16 (10, 23) | 18 (12, 27) |
| **% hospital turnround times >30 mins** | 8 (4, 16) | 8 (5, 16) | 8 (4, 16) | 6 (2, 15) | 7 (2, 16) | 6 (2, 15) | 10 (4, 19) | 10 (5, 20) | 10 (4, 19) |
| **Clinician length of service (years)** | 6.9  (2.5, 14.9) | 6.4  (2.4, 14.4) | 6.8  (2.5, 14.9) | 6  (4, 15) | 5  (3, 11) | 6  (4, 14) | NA | NA | NA |
| **Patient age (years)** | 63 (39, 80) | 67 (46, 80) | 64 (40, 80) | 59 (35, 79) | 70 (51, 82) | 61 (36, 79) | 65 (41, 81) | 71 (52, 82) | 66 (43, 81) |
| **First NEWS2** | 1  (0, 4) | 5  (2, 8) | 2 (0, 4) | 2 (0, 4) | 6. (3, 9) | 2 (0, 4) | 1  (0, 3) | 6  (2, 9) | 2 (0, 4) |
| **Last NEWS2** | 1  (0, 3) | 5  (2, 8) | 1  (0, 4.) | NA | NA | NA | 1  (0, 3) | 5 (2, 7) | 1 (0, 3) |

^1^Median (IQR)
