# Supplementary material for: What factors predict ambulance prealerts to the emergency department? Retrospective observational study from three UK ambulance services
Source: BMJ Open. 2025 Mar 7;15(3):e097122. doi: 10.1136/bmjopen-2024-097122 (PMC11891521; doi:10.1136/bmjopen-2024-097122)
Supplement: online supplemental file 4 [file bmjopen-15-3-s004.docx]

| **Table 2:** Summary table of ambulance transports to hospital stratified by pre-alert. | | | | |
| --- | --- | --- | --- | --- |
| **Characteristic** | **Site 1 Pre-alert / total (%)** | **Site 2 Pre-alert / total (%)** | **Site 3 Pre-alert / total (%)** | **Total Pre-alert / total (%)** |
| **Overall** | 60,549/ 413,140  (14.7%) | 51,142//623,325  (8.2%) | 31,104 /326,809  (9.5%) | 142,795/1,363,274  (10.5%) |
| **Simplified clinician role** |  |  |  |  |
| Paramedic | 46,665/305,549  (15%) | 39,342/485,978  (8.1%) | 21,302/211,206  (10%) | 107,309/1,002,733  (11%) |
| Non-paramedic | 13,884/107,591  (13%) | 11,800/137,347  (8.6%) | 9,802/115,603  (8.5%) | 35,486/360,541  (9.8%) |
| **Patient sex** |  |  |  |  |
| Male | 31,550/196,484  (16%) | 26,922/298,979  (9.0%) | 16,263/157,058  (10%) | 74,735/652,521  (11%) |
| Female | 27,871/210,636  (13%) | 23,835/319,200  (7.5%) | 14,764/168,699  (8.8%) | 66,470/698,535  (9.5%) |
| Not Specified | 0/0 (%) | 385/5,146  (7.5%) | 53/691  (7.7%) | 438/5,837  (7.5%) |
| Transgender | 15/163 (9.2%) | 0/0 (%) | 22/355 (6.2%) | 37/518 (7.1%) |
| **Working Impression** |  |  |  |  |
| Sepsis | 6,705/10,138  (66%) | 11,402/20,679  (55%) | 3,372/4,004  (84%) | 21,479/34,821  (62%) |
| Unspecified medical condition | 5,668/62,620  (9.1%) | 8,673/165,959  (5.2%) | 2,470/48,208  (5.1%) | 16,811/276,787  (6.1%) |
| Acute Stroke | 5,881/8,308  (71%) | 6,417/13,812  (46%) | 2,571/5,663  (45%) | 14,869/27,783  (54%) |
| Other working impression | 5,791/71,923  (8.1%) | 3,848/90,264  (4.3%) | 2,134/54,066  (3.9%) | 11,773/216,253  (5.4%) |
| COVID-19 | 3,525/13,942  (25%) | 4,481/25,550  (18%) | 928/4,285  (22%) | 8,934/43,777  (20%) |
| Respiratory problem | 5,839/26,628  (22%) | 676/6,479 (10%) | 2,070/15,144  (14%) | 8,585/48,251  (18%) |
| Arrhythmia | 2,504/6,740  (37%) | 1,530/10,335  (15%) | 1,970/8,069  (24%) | 6,004/25,144  (24%) |
| Lower respiratory tract infection | 926/5,059  (18%) | 2,321/19,489  (12%) | 2,532/13,287  (19%) | 5,779/37,835 (15%) |
| Cardiac problem | 1,919/21,419  (9.0%) | 701/22,097  (3.2%) | 2,687/34,571  (7.8%) | 5,307/78,087  (6.8%) |
| Trauma: other | 1,461/13,590  (11%) | 1,677/70,944  (2.4%) | 1,776/34,630  (5.1%) | 4,914/119,164  (4.1%) |
| Convulsion | 2,516/11,135  (23%) | 1,052/8,651  (12%) | 407/3,283  (12%) | 3,975/23,069  (17%) |
| STEMI | 1,728/2,059  (84%) | 1,517/3,025  (50%) | 441/1,122  (39%) | 3,686/6,206  (59%) |
| Head injury | 1,288/12,352  (10%) | 1,286/31,482  (4.1%) | 722/13,337  (5.4%) | 3,296/57,171  (5.8%) |
| Overdose | 1,762/11,565  (15%) | 284/4,807  (5.9%) | 917/9,463  (9.7%) | 2,963/25,835  (11%) |
| COPD | 1,358/4,089  (33%) | 867/4,921  (18%) | 630/3,544  (18%) | 2,855/12,554  (23%) |
| Major trauma | 1,580/2,526  (63%) | 437/3,276  (13%) | 311/958  (32%) | 2,328/6,760  (34%) |
| Neurological problem | 673/2,664  (25%) | 0/13  (0%) | 1,554/15,514  (10%) | 2,227/18,191  (12%) |
| Unspecified infection | 326/3,108  (10%) | 429/7,010  (6.1%) | 1,465/11,482  (13%) | 2,220/21,600  (10%) |
| Acute abdominal | 1,022/26,186 (3.9%) | 381/38,647  (1.0%) | 672/24,946  (2.7%) | 2,075/89,779  (2.3%) |
| Metabolic problem | 868/3,898  (22%) | 422/4,369  (9.7%) | 678/3,757  (18%) | 1,968/12,024  (16%) |
| Total of others | 7,209/93,191  (7.7%) | 2,741/71,516  (3.8%) | 797/17,476  (4.6%) | 10,747/182,183  (5.9%) |
| Any RCEM Pre-alert criteria triggered | 40,437/98,149  (41%) | 40,250/145,989  (28%) | 22,536/80,461  (28%) | 103,223/324,599  (32%) |
| Any RCEM physiological criteria triggered | 28,990/79,956  (36%) | 29,027/117,873  (25%) | 18,911/71,907  (26%) | 76,928/269,736  (29%) |
| Any RCEM non-physiological criteria triggered | 22,566/34,267  (66%) | 24,722/57,871 (43%) | 8,658/17,209  (50%) | 55,946/109,347  (51%) |
| **Triage Category** |  |  |  |  |
| 1 | 11,423/38,678 (30%) | 7,285/47,861 (15%) | 12/382 (3.1%) | 18,720/86,921 (22%) |
| 2 | 41,974/277,608 (15%) | 34,159/334,151  (10%) | 27,409/257,389  (11%) | 103,542/869,148  (12%) |
| 3 | 6,701/85,055  (7.9%) | 8,363/199,264  (4.2%) | 3,543/65,668  (5.4%) | 18,607/349,987  (5.3%) |
| 4 | 347/10,391  (3.3%) | 247/7,780  (3.2%) | 15/1,005  (1.5%) | 609/19,176  (3.2%) |
| 5 | 104/1,408  (7.4%) | 13/408  (3.2%) | 125/2,365  (5.3%) | 242/4,181  (5.8%) |
| **Hospital Department** |  |  |  |  |
| ED | 55,703/376,831  (15%) | 48,263/574,222  (8.4%) | 27,513/280,214  (9.8%) | 131,479/1,231,267  (11%) |
| Acute e.g. pPCI | 3,070/4,269  (72%) | 1,952/5,028  (39%) | 361/2,676  (13%) | 5,383/11,973  (45%) |
| Unknown | 22/1,355  (1.6%) | 632/24,326  (2.6%) | 2,518/28,691 (8.8%) | 3,172/54,372  (5.8%) |
| Ward | 1,235/14,286  (8.6%) | 295/17,767  (1.7%) | 93/3,535  (2.6%) | 1,623/35,588  (4.6%) |
| Same day e.g. IUC | 519/16,399  (3.2%) | 0/1,982  (0%) | 619/11,693  (5.3%) | 1,138/30,074  (3.8%) |

**Table 4:** Summary of multivariable logistic regression following LASSO variable selection for ambulance service pre-alert for sites 1 and 2.

| **Term** | **Odds Ratio (95%CI)** |
| --- | --- |
| Newly qualified Paramedic | 1.3 (1.26–1.34) |
| Paramedic | 1.11 (1.08–1.13) |
| Senior clinician | 1.09 (1.03–1.15) |
| Male clinician | 0.98 (0.96–1) |
| Proportion of hospital turnarounds exceeding 30 minutes | 1.74 (1.6–1.9) |
| Patient age | 1.01 (1.01–1.01) |
| Male patient | 1.24 (1.22–1.26) |
| Patient presentation meets RCEM non-physiological pre-alert criteria | 15.85 (15.52–16.18) |
| Site 1 ambulance service | 1.94 (1.86–2.02) |
| ED1 | 1.12 (1.04–1.2) |
| ED2 | 1.57 (1.5–1.64) |
| ED3 | 0.78 (0.71–0.85) |
| ED4 | 1.43 (1.34–1.51) |
| ED5 | 0.38 (0.18–0.7) |
| ED6 | 0.84 (0.77–0.92) |
| ED7 | 0.53 (0.47–0.59) |
| ED8 | 1.21 (1.15–1.27) |
| ED9 | 0.82 (0.75–0.89) |
| ED10 | 0.92 (0.86–0.98) |
| ED11 | 0.69 (0.64–0.74) |
| ED12 | 1.2 (1.13–1.26) |
| ED13 | 1.13 (1.05–1.21) |
| ED14 | 0.81 (0.76–0.86) |
| MTC1 | 1.3 (1.25–1.36) |
| MTC2 | 1.1 (0.98–1.23) |
| MTC3 | 1.66 (1.58–1.74) |
| ED15 | 1.14 (1.08–1.2) |
| MTC4 | 1.51 (1.43–1.58) |
| MTC5 | 0.89 (0.85–0.93) |
| Other | 0.56 (0.5–0.63) |
| ED17 | 1.8 (1.73–1.88) |
| ED18 | 0.87 (0.8–0.93) |
| ED19 | 1.18 (1.12–1.25) |
| ED20 | 0.74 (0.68–0.8) |
| MTC6 | 0.81 (0.77–0.85) |
| ED21 | 0.86 (0.81–0.91) |
| ED22 | 0.84 (0.7–1) |
| MTC7 (not highlighted in table 4 of main document due to CI) | 3.18 (0.66–10.82) |
| MTC8 | 0.9 (0.85–0.96) |
| ED23 | 0.78 (0.73–0.84) |
| ED24 | 1.52 (1.43–1.61) |
| ED25 | 2.2 (2.11–2.3) |

ED prefix denotes emergency department, MTC denotes tertiary hospital designated as a major trauma centre.
