# Supplementary material for: What factors predict ambulance prealerts to the emergency department? Retrospective observational study from three UK ambulance services
Source: BMJ Open. 2025 Mar 7;15(3):e097122. doi: 10.1136/bmjopen-2024-097122 (PMC11891521; doi:10.1136/bmjopen-2024-097122)
Supplement: Supplementary file 5 [file bmjopen-2024-097122.reviewer_comments.pdf]

PEER REVIEW HISTORY

BMJ Open publishes all reviews undertaken for accepted manuscripts. Reviewers are asked to complete a checklist review form and are provided with free text boxes to elaborate on their assessment. These free text comments are reproduced below.

ARTICLE DETAILS

Title (Provisional)

What factors predict ambulance pre-alerts to the emergency department? Restrospective Observational study from three UK ambulance services.

Authors

Pilbery, Richard; Sampson, Fiona C; Herbert, Esther; Goodacre, Steve; Bell, Fiona; Spaight, Rob; Rosser, Andy; Webster, Peter; Millins, Mark; Pountney, Andrew; Coster, Joanne; Long, Jaqui; O'Hara, Rachel; Foster, Alexis; Miles , Jamie; Turner, Janette; Boyd, Aimee

---

VERSION 1 - REVIEW

**Reviewer**	**1**
**Name**	**Tuli, Wagari**
**Affiliation**	**Addis Ababa University, Emergency Medicine and Critical Care**
**Date**	**19-Dec-2024**
**COI**	**None**

Wagari Tuli (Assistant professor) at Addis Ababa University, college of Health Sciences

Title: What factors predict ambulance pre-alerts to the emergency department? Analysis of routine data from three UK ambulance services.

Manuscript ID: bmjopen-2024-097122

First of all, I would like to appreciate editorial team of BMJ Open for inviting me to review this manuscript. I also need to appreciate the authors for doing this important title so that I want to put my minor comments as follow:

1. Title: line 2- UK- use full name in the title rather than abbreviation

2. Strengths and limitations of the study- line 67-…1.3m ambulance conveyances… 1.3m(write it as million because it seems meter)

3. Line 77: Departments (ED)- this is already defined under abstract, no need to repeat, simply use ED.

4. Line 110: define abbreviation of NHS here(first) and remove it that you defined it on line 116.

5. Methods: line 114- let say Methods and Materials. Your Methods is not clear: e.g. How many ambulance services are there in UK? what are they? (their names), who are your data collectors and how did you recruit them? Specify, and do you think this study generalises the all-ambulance services in the UK (I mean by doing on only 3 ambulance services)?

6. Line 116: the same for UK (you defined it here after you used it many times in documents above methods)

7. Line 136 &7: not clear about SITREP data.23; clarify it

8. Table 1: line 145- it is better if you tell us some picture of the sites you mentioned as site 1,2&3 under setting as part of your study area (look at the above comments under methods and let you match both comments).

9. Table 2&3: line 194 & 5: their titles are similar. Why did you use similar title while the entire contents are different?

10. Table 4: line 206: talks about multivariable logistic regression but on the table, it is too difficult to identify which factor has a significant association with pre-alert because you didn't mention the p values.

11. Ethics approval: 427: It needs detail explanation rather than putting only reference number; the process, participants 'consent if needed and privacy of the participants.

12. Additional file 2: also, it is better if you add p-values for all ORs

13. Lastly, try to distribute your text evenly between the margins

---

**Reviewer**	**2**
**Name**	**Rente, Maria**
**Affiliation**	**Alentejo Coast Local Health Unit, Emergency Department**
**Date**	**22-Dec-2024**
**COI**	**None**

- The article title should include the study design: retrospective observational study.

2. In the abstract, the objective of the study should be clear, as presented in the introduction ("To understand whether there were clinician, patient and/or hospital factors that influenced pre-alert practice beyond the patient's presenting complaint."), and the main research question should be covered ("What factors predict ambulance pre-alerts to the emergency department?"). According to the Submission guidelines for original research and based on the CONSORT extension for abstracts, the design should look like this (design: retrospective observational study), followed by setting ("three UK ambulance services").

Participants ("1,363,274 patients conveyed to ED", clear definitions of selection, entry and exclusion criteria "We excluded patients under 16 years of age due to different physiological/ NEWS2 thresholds."). Primary and secondary outcome measures.

- Strengths and limitations of this study (Correct the – Line 64).

- Funding statement (Add statement – Line 439). Change to "This work was supported by National Institute for Health and Care Research grant number NIHR HS&DR 131293.".

- Competing interests statement (Add statement – Line 437). Change to "None declared", as mentioned in BMJ Author Hub.

- Author's contribution: As mentioned in ICMJE, change to "Substantial contributions to the conception or design of the work; or the acquisition, analysis, or interpretation of data for the work; AND Drafting the work or reviewing it critically for important intellectual content; AND Final approval of the version to be published; AND Agreement to be accountable for all aspects of the work in ensuring that questions related to the accuracy or integrity of any part of the work are appropriately investigated and resolved.".

- Insert Patient consent form: Not applicable.

- Insert Data sharing statemen: Not applicable.

- Reformulate the discussion section in order to respond to: a statement of the principal findings; strengths and weaknesses of the study; strengths and weaknesses in relation to other studies, discussing important differences in results; the meaning of the study: possible explanations and implications for clinicians and policymakers; and unanswered questions and future research.

8. It would be desirable for the bibliographical references (30) to be representative of the scientific evidence produced and indexed in the last 5 years (80%) or more than 6 years (20%). However, the scarcity of current evidence on the subject demonstrates the importance of carrying out this and future studies on the subject in question.

- Line 36: Delete the space after the word variation.

- Line 67: Delete the space after the word conveyances.

- Line 70: Suggestion, select one, time or period (Conciseness – More concise language would be clearer for reader).

- Line 72: Suggestion, delete "in order" (Conciseness – More concise language would be clearer for reader).

- Line 118: Delete the space after the word technicians.

- Line 119: Suggestion, replace "In the event that" with If (Conciseness – More concise language would be clearer for reader).

- Line 151: Suggestion, delete “in order” (Conciseness – More concise language would be clearer for reader).

- Line 155: Suggestion, delete “in order” (Conciseness – More concise language would be clearer for reader).

- Line 156: Suggestion, I think it's pre-alert.

- Line 174: Suggestion, plural, groups.

- Line 175: Suggestion, grammar, double-check that you’re sticking to singular or plural, was or were.

- Line 186: Suggestion, I think it's pre-alert.

- Line 187: Correction, and.

- Line 198: Separate age or

- Line 213: Suggestion, grammar, double-check whether the noun is singular or plural, increased odd of a pre-alert.

- Line 232: Suggestion, I think it's pre-alert.

- Line 296/297: Suggestion, select one, time or period (Conciseness – More concise language would be clearer for reader).

- Line 303/304: Suggestion, I think it's pre-alert.

The reviewer provided a marked copy with additional comments. Please contact the publisher for full details.

Reviewer 3

Name Hanaki, Nao

Affiliation Osaka University School of Medicine Graduate School of Medicine, Department of Public Health

Date 10-Jan-2025

COI None

Thank you to authors for submitting an interesting paper.

This paper studies factors predict ambulance pre-alerts to the emergency department. It uses large-scale data and has some ingenious analysis techniques, such as using a LASSO model to select variables for multivariate analysis.

Several minor revisions are required for the paper to be accepted.

My comments are listed below. I hope that my comments will help to make the paper better.

#1

Some abbreviations are missing in the initial description, please mention SITREP data.23 in Page 5 Line 137, ONS (Office for National Statistics) in Page 6 Table 1.

#2

Page 6 Table 1

Table 1 contains an explanation of the variables, but the explanation of the categorization of the variables is insufficient. Since there are many variables handled, I think it would be one way to describe them in an Additional File.

Some of the variables that caught my attention are as follows.

Hospital characteristics

The results show characteristics such as ICU, but please also state which characteristics you divided by in the Method.

Length of experience

Did years of experience enter as a variable? Please write so that it is clear which content was actually treated as a variable.

NEWS2

NEWS2 is entered twice, but at what point in time was this? If it is fixed, such as when the injured person first came into contact with the patient or when the patient was carried into the ambulance, please write it specifically. Also, please explain whether the timing of entry is the same for all ambulance teams and whether there is a fixed protocol.

#3

Sensitivity Analysis

The fact that a sensitivity analysis was performed is described in the Method section on page 7, but I was unable to find any information on the results. It is mentioned in the Discussion section, but where is it written in the Results section? The STROBE checklist says P17, but in the PDF file I checked, Page 17 was the page with the references.

Please make it clear so that first-time readers can check the results.

Reviewer 4

Name Johnston, Aaron

Affiliation University of Calgary Cumming School of Medicine, Distributed Learning and Rural initiatives

Date 10-Jan-2025

COI None

This is an interesting paper and adds to the knowledge base around prehospital medicine. I have a few questions about the paper, but suspect that my larger questions may not be answerable through your data set. I have therefore chosen accept rather than revise, though if any of the questions I raise are answerable I think that the answers would strengthen the paper. There are 2 editorial/organizational suggestions at the bottom which I would request are acted on.

1. Is it possible to do a subgroup analysis of the pre-alerts with unspecified medical condition and other clinical impression? It seems like there could be a lot of variability hidden within this group.

2. The culture at the interface of the transition between prehospital and hospital care is mentioned in the discussion. Could the paramedics knowledge about the cultural expectations of the hospital site they are bringing the patient to account for the variability between hospitals?

3. Does the data allow you to determine of there were high and low pre-alert activators? Are there individuals whose practice patterns are outliers?

4. Line 183-184, Do paramedics and non paramedics transport different types of patients?

5. Line 187, Was COVID a specified per-alert during the period of 'lockdown'? Is there a temporal trend in COVID pre-alert?

6. Did you discover any temporal trends in the data? Differences between day/night, seasonal variation?

A few smaller issues:

1. Line 60 requires revision for clarity. In fact illness severity and clinical impression do impact pre-alert decision, but factors beyond this do as well. Currently it reads as if illness severity and clinical impression do not play a role.

2. Please define NEWS2 with first use on line 127. The paper would benefit from a single line here describing NEWS2, I would suggest moving lines 146 and 147 to this point in the paper.

VERSION 1 - AUTHOR RESPONSE

	Reviewer comment	Our response
1	Reviewer: 1<br>Mr. Wagari Tuli, Addis Ababa University, Addis Ababa University College of Health Sciences<br>Comments to the Author:<br><br>First of all, I would like to appreciate editorial team of BMJ Open for inviting me to review this manuscript. I also need to appreciate the authors for doing this important title so that I want to put my minor comments as follow:	

2	1. Title: line 2- UK- use full name in the title rather than abbreviation	We have amended this.
3	2. Strengths and limitations of the study- line 67- …1.3m ambulance conveyances… 1.3m(write it as million because it seems meter)	We have amended this.
4	3. Line 77: Departments (ED)- this is already defined under abstract, no need to repeat, simply use ED.	We are leaving this as it stands as this is the first time the abbreviation has been mentioned in the body of the paper.
5	4. Line 110: define abbreviation of NHS here(first) and remove it that you defined it on line 116.	We have amended this.
6	5. Methods: line 114- let say Methods and Materials. Your Methods is not clear: e.g. How many ambulance services are there in UK? what are they? (their names), who are your data collectors and how did you recruit them? Specify, and do you think this study generalises the all-ambulance services in the UK (I mean by doing on only 3 ambulance services)?	We agree that further details here would be useful. We have added in further details about the characteristics of the ambulance services from whom we collected data. We have also added in a line to the limitations section where we discuss this (p14).<br>The data collected was routine data so was not specifically collected for the purposes of this study.
7	6. Line 116: the same for UK (you defined it here after you used it many times in documents above methods)	We have amended this.
8	7. Line 136 &7: not clear about SITREP data.23; clarify it	We have amended this. – this is also referenced so that the reader can view the source.
9	8. Table 1: line 145- it is better if you tell us some picture of the sites you mentioned as site 1,2&3 under setting as part of your study area (look at the above comments under methods and let you match both comments).	We have amended this - see line 6.
10	9. Table 2&3: line 194 & 5: their titles are similar. Why did you use similar title while the entire contents are different?	Thank you for pointing this out. The two tables presented different categories of variables but we had not explained this. We have now amended the text.
11	10. Table 4: line 206: talks about multivariable logistic regression but on the table, it is too difficult to identify which factor has a significant association with pre-alert because you didn't mention the p values.	The model in Table 4 was fitted using LASSO regression, a method of variable selection which does not use statistical significance to build the model. We also feel that presenting p-values for this model would be misleading without correction for multiplicity, as this was an exploratory analysis with no formal hypothesis testing p-values would be inappropriate. However, we have provided the 95% confidence intervals which do indicate statistical significance whilst also showing the uncertainty around the estimates of the coefficients in the model.
12	11. Ethics approval: 427: It needs detail explanation rather than putting only reference number; the process, participants 'consent if needed and privacy of the participants.	We have provided the reference number of the ethical approval and name of the NHS Research Ethics Committee who provided the approval.
13	12. Additional file 2: also, it is better if you add p-values for all ORs	See response 11

14	13. Lastly, try to distribute your text evenly between the margins	We have amended this.
15	Reviewer: 2<br>Miss Maria Rente, Alentejo Coast Local Health Unit<br>Comments to the Author:	
16	The article title should include the study design: retrospective observational study.	We have amended this.
17	2. In the abstract, the objective of the study should be clear, as presented in the introduction ("To understand whether there were clinician, patient and/or hospital factors that influenced pre-alert practice beyond the patient's presenting complaint."), and the main research question should be covered ("What factors predict ambulance pre-alerts to the emergency department?"). According to the Submission guidelines for original research and based on the CONSORT extension for abstracts, the design should look like this (design: retrospective observational study), followed by setting ("three UK ambulance services"). Participants ("1,363,274 patients conveyed to ED", clear definitions of selection, entry and exclusion criteria "We excluded patients under 16 years of age due to different physiological/ NEWS2 thresholds."). Primary and secondary outcome measures.	We have amended the abstract to incorporate some of these suggestions but have not been able to incorporate all suggestions due to the word limit for the abstract. We have used the same wording in the objective for the abstract as we used within the introduction.
18	- Strengths and limitations of this study (Correct the – Line 64).	We have amended this.
19	- Funding statement (Add statement – Line 439). Change to "This work was supported by National Institute for Health and Care Research grant number NIHR HS&DR 131293.".	We have amended this.
20	- Competing interests statement (Add statement – Line 437). Change to "None declared", as mentioned in BMJ Author Hub.	We have amended this.
21	- Author's contribution: As mentioned in ICMJE, change to "Substantial contributions to the conception or design of the work; or the acquisition, analysis, or interpretation of data for the work; AND Drafting the work or reviewing it critically for important intellectual content; AND Final approval of the version to be published; AND Agreement to be accountable for all aspects of the work in ensuring that questions related to the accuracy or integrity of any part of the work are appropriately investigated and resolved.".	We have added in the ICMJE statement as requested.
22	Insert Patient consent form: Not applicable.	NA

23	- Insert Data sharing statemen: Not applicable.	NA
24	- Reformulate the discussion section in order to respond to: a statement of the principal findings; strengths and weaknesses of the study; strengths and weaknesses in relation to other studies, discussing important differences in results; the meaning of the study: possible explanations and implications for clinicians and policymakers; and unanswered questions and future research.	We feel that we have structured our discussion in the way suggested, although we have not explicitly labelled each section of the discussion as such because we have structured the discussion according to the journal style. We think that we have covered each of these areas.
25	8. It would be desirable for the bibliographical references (30) to be representative of the scientific evidence produced and indexed in the last 5 years (80%) or more than 6 years (20%). However, the scarcity of current evidence on the subject demonstrates the importance of carrying out this and future studies on the subject in question.	We agree and hope that publishing our study findings will provide more recent literature on the topic.
26	- Line 36: Delete the space after the word variation.	We have amended this.
27	- Line 67: Delete the space after the word conveyances.	We have amended this.
28	- Line 70: Suggestion, select one, time or period (Conciseness – More concise language would be clearer for reader).	Although we agree that this could be more concise, we feel that it is important to provide both the time period and the reference to the fact that this was the second period of Covid-19 lockdown.
29	- Line 72: Suggestion, delete "in order" (Conciseness – More concise language would be clearer for reader).	We have amended this.
30	- Line 118: Delete the space after the word technicians.	We have amended this.
31	- Line 119: Suggestion, replace "In the event that" with If (Conciseness – More concise language would be clearer for reader).	We have amended this.
32	- Line 151: Suggestion, delete "in order" (Conciseness – More concise language would be clearer for reader).	We have amended this.
33	- Line 155: Suggestion, delete "in order" (Conciseness – More concise language would be clearer for reader).	We have amended this.
34	- Line 156: Suggestion, I think it's pre-alert.	We can't see the word 'pre-alert' missing from this sentence.
35	- Line 174: Suggestion, plural, groups.	There was one PPI group. We have clarified some wording on line 175 as this may be where the confusion lies.
36	- Line 175: Suggestion, grammar, double-check that you're sticking to singular or plural, was or were.	We have checked this grammar to ensure we are using the appropriate terms. .
37	- Line 186: Suggestion, I think it's pre-alert.	Pre-alert should be plural here.
38	- Line 187: Correction, and.	We have amended this.
39	- Line 198: Separate age or	We have amended this.

40	- Line 213: Suggestion, grammar, double-check whether the noun is singular or plural, increased odd of a pre-alert.	We have used 'odds' in this case because of it being the term commonly used to describe probability.
41	- Line 232: Suggestion, I think it's pre-alert.	Pre-alert should be plural here.
42	- Line 296/297: Suggestion, select one, time or period (Conciseness – More concise language would be clearer for reader).	We feel that it is important to retain both descriptions of the time period (i.e. the months, and the fact that this was the covid-19 lockdown).
43	- Line 303/304: Suggestion, I think it's pre-alert.	We cannot see the word 'pre-alert' missing from this section.
44		
45	Reviewer: 3<br>Dr. Nao Hanaki, Osaka University School of Medicine Graduate School of Medicine<br>Comments to the Author:<br>Thank you to authors for submitting an interesting paper.<br><br>This paper studies factors predict ambulance pre-alerts to the emergency department. It uses large-scale data and has some ingenious analysis techniques, such as using a LASSO model to select variables for multivariate analysis.<br><br>Several minor revisions are required for the paper to be accepted.<br><br>My comments are listed below. I hope that my comments will help to make the paper better.	
46	#1 Some abbreviations are missing in the initial description, please mention SITREP 20data.23 in Page 5 Line 137, ONS (Office for National Statistics) in Page 6 Table 1.	We have amended this.
47	#2<br>Page 6 Table 1<br>Table 1 contains an explanation of the variables, but the explanation of the categorization of the variables is insufficient. Since there are many variables handled, I think it would be one way to describe them in an Additional File.<br><br>Some of the variables that caught my attention are as follows.<br><br>Hospital characteristics<br>The results show characteristics such as ICU, but please also state which characteristics you divided by in the Method.<br><br>Length of experience<br>Did years of experience enter as a variable? Please write so that it is clear which content was	We have revised Table 1 to improve clarity. An additional file outlining the RCEM pre-alert criteria is included in the submission.<br><br>Hospital characteristics: we are not clear what the reviewer means here. We cannot find a mention of ICU within our paper.<br><br>Length of experience: We have amended table 1 to make this clearer.<br><br>NEWS2: Since we utilised routine data, it was not possible to specify timings for when NEWS2 scores should be recorded. The 'first NEWS2 score' is the first documented NEWS2 score in the observations section of the electronic patient record and the 'last NEWS2 score' is the final documented NEWS2 score

	actually treated as a variable.<br><br>NEWS2<br>NEWS2 is entered twice, but at what point in time was this? If it is fixed, such as when the injured person first came into contact with the patient or when the patient was carried into the ambulance, please write it specifically. Also, please explain whether the timing of entry is the same for all ambulance teams and whether there is a fixed protocol.	in the observations section. We are unable to state exactly when this took place.
48	#3<br>Sensitivity Analysis<br>The fact that a sensitivity analysis was performed is described in the Method section on page 7, but I was unable to find any information on the results. It is mentioned in the Discussion section, but where is it written in the Results section? The STROBE checklist says P17, but in the PDF file I checked, Page 17 was the page with the references.<br>Please make it clear so that first-time readers can check the results.	On reflection the team felt that the sensitivity analysis did not add to the message of the paper and since it was a post-hoc analysis (not pre-specified) the decision has been made to remove the reference to it.
	Reviewer: 4<br>Dr. Aaron Johnston, University of Calgary Cumming School of Medicine<br>Comments to the Author:<br>This is an interesting paper and adds to the knowledge base around prehospital medicine. I have a few questions about the paper, but suspect that my larger questions may not be answerable through your data set. I have therefore chosen accept rather than revise, though if any of the questions I raise are answerable I think that the answers would strengthen the paper. There are 2 editorial/organizational suggestions at the bottom which I would request are acted on.	
49	1. Is it possible to do a subgroup analysis of the pre-alerts with unspecified medical condition and other clinical impression? It seems like there could be a lot of variability hidden within this group.	We could undertake this analysis as part of a further study. However, we have only reported the analyses that we specified within our original statistical analysis plan (as submitted to our funder).
50	2. The culture at the interface of the transition between prehospital and hospital care is mentioned in the discussion. Could the paramedics knowledge about the cultural expectations of the hospital site they are bringing the patient to account for the variability between hospitals?	This was a finding we identified in the qualitative part of our study (reported elsewhere, now also referenced within this paper). We have added in another sentence within the paper linking to findings from one of our linked qualitative papers, suggesting that knowledge of hospital expectations influenced decision-making.

51	3. Does the data allow you to determine of there were high and low pre-alert activators? Are there individuals whose practice patterns are outliers?	We did not include this question within our initial analysis. We may be able to explore pre-alert rates by clinician (adjusted for skillmix) but this is not within the scope for this study and was not specified within our funded statistical analysis plan.
52	4. Line 183-184, Do paramedics and non paramedics transport different types of patients?	Ambulance crews usually include a paramedic (registered healthcare professional) and a non-paramedic. However, in some cases ambulances can be crewed by a paramedic and e.g. a newly qualified paramedic under supervision, or two non-paramedics who may be specifically assigned to low acuity transport for example the movement of clinical stable patients between healthcare settings. Other combinations are of course possible, but the level of qualification of the crew changes the scope of practice in terms of decision making and may be taken into consideration in dispatching an ambulance to a particular presentation or category of call.<br>However, we cannot consistently state that paramedics and non-paramedics transport different types of patient.
53	5. Line 187, Was COVID a specified per-alert during the period of 'lockdown'? Is there a temporal trend in COVID pre-alert?	Yes - suspected Covid-19 was a diagnosis code used during this period (see table 2). We did not include temporal trends in final analysis although we did include 'final hour of shift' due to the suggestion that ambulance clinicians may be more likely to pre-alert during the final hour of shift.
54	6. Did you discover any temporal trends in the data? Differences between day/night, seasonal variation?	We did look at temporal trends in the data initially but did not have any rationale to include these within the analysis.
55	A few smaller issues:<br>1. Line 60 requires revision for clarity. In fact illness severity and clinical impression do impact pre-alert decision, but factors beyond this do as well. Currently it reads as if illness severity and clinical impression do not play a role.	We agree and have added text to clarify this.
56	2. Please define NEWS2 with first use on line 127. The paper would benefit from a single line here describing NEWS2, I would suggest moving lines 146 and 147 to this point in the paper.	We have added an explanation of NEWS2 to the introduction as suggested. However, we feel that the full, referenced definition of NEWS2 is more appropriate in table 1 than in the introduction. When we tried to move it to the introduction we felt that it broke the flow of the sentence.
